# Supplementary material for: Novel Nongenomic Signaling by Glucocorticoid May Involve Changes to Liver Membrane Order in Rainbow Trout
Source: PLoS One. 2012 Oct 8;7(10):e46859. doi: 10.1371/journal.pone.0046859 (PMC3466178; doi:10.1371/journal.pone.0046859)
Supplement: Figure S1 — Effect of cortisol, RU486, benzyl alcohol & DMSO on membrane fluidity. Anisotropy of isolated hepatic membranes with cortisol (1 µM) RU486 (1 µM) combination treatment (RU+CORT; both 1 µM) benzyl alcohol (BOH; 5 mM), dimethyl sulphoxide (DMSO, 2% v/v) or without (control) at both 4°C and 23°C. Values are shown as % control and bars represent means ± S.E.M. (N = 3–9 independent membrane preparations). (DOCX) [file pone.0046859.s001.docx]

**Supporting Information (SI)**

**Figure S1. Effect of cortisol, RU486, benzyl alcohol & DMSO on membrane fluidity.**

Anisotropy of isolated hepatic membranes with cortisol (1 µM) RU486 (1 µM) combination treatment (RU+CORT; both 1 µM) benzyl alcohol (BOH; 5mM), dimethyl sulphoxide (DMSO, 2% v/v) or without (control) at both 4^o^C and 23^o^C. Values are shown as % control and bars represent means ± S.E.M. (N = 3-9 independent membrane preparations).

Figure S1.
